# Supplementary figures and images for: The effect of experiential learning interventions on physical activity outcomes in children: A systematic review
Source: PLoS One. 2023 Nov 30;18(11):e0294987. doi: 10.1371/journal.pone.0294987 (PMC10688861; doi:10.1371/journal.pone.0294987)

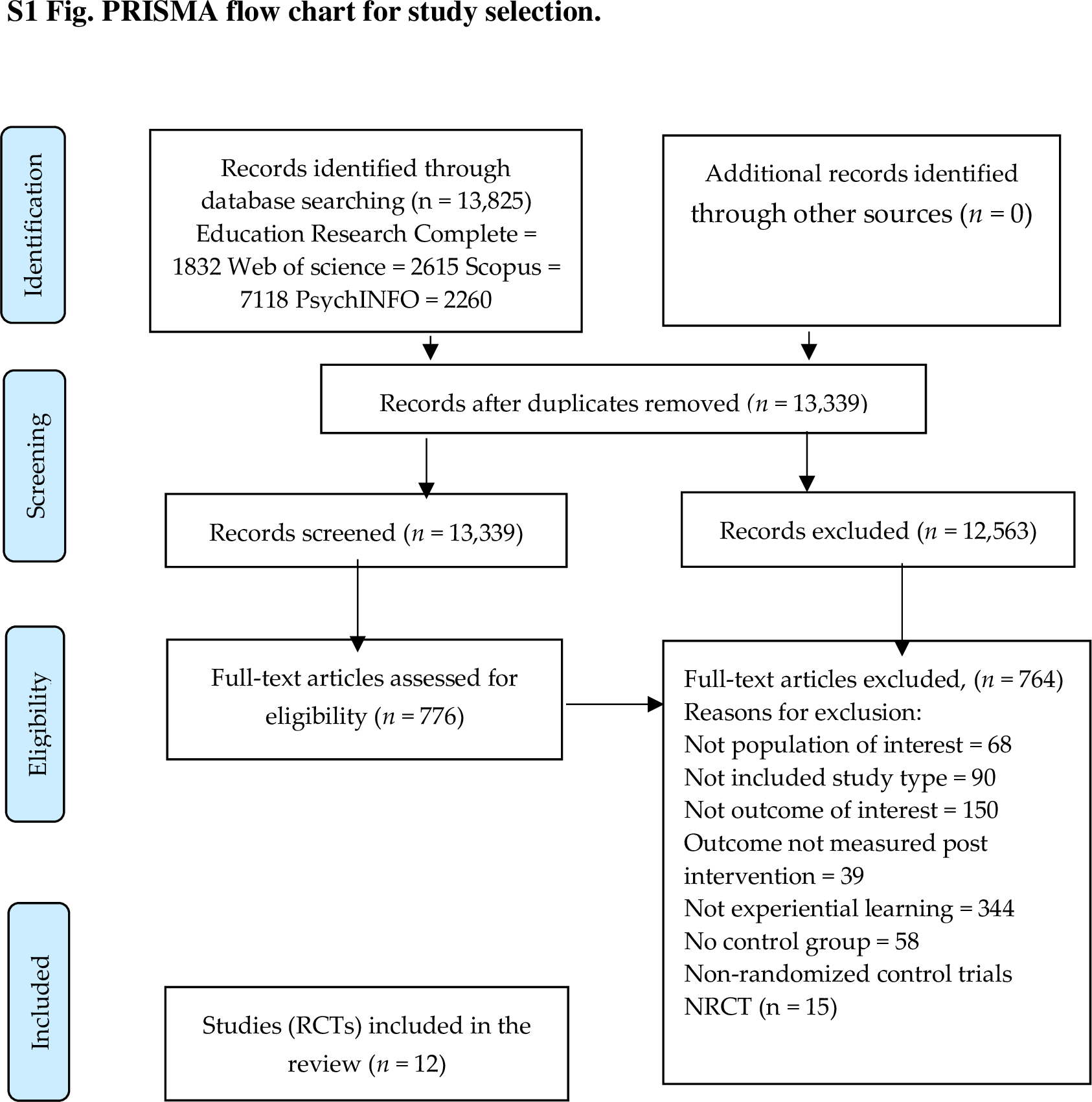

Supplement: S1 Fig — (TIF) [file pone.0294987.s001.tif]

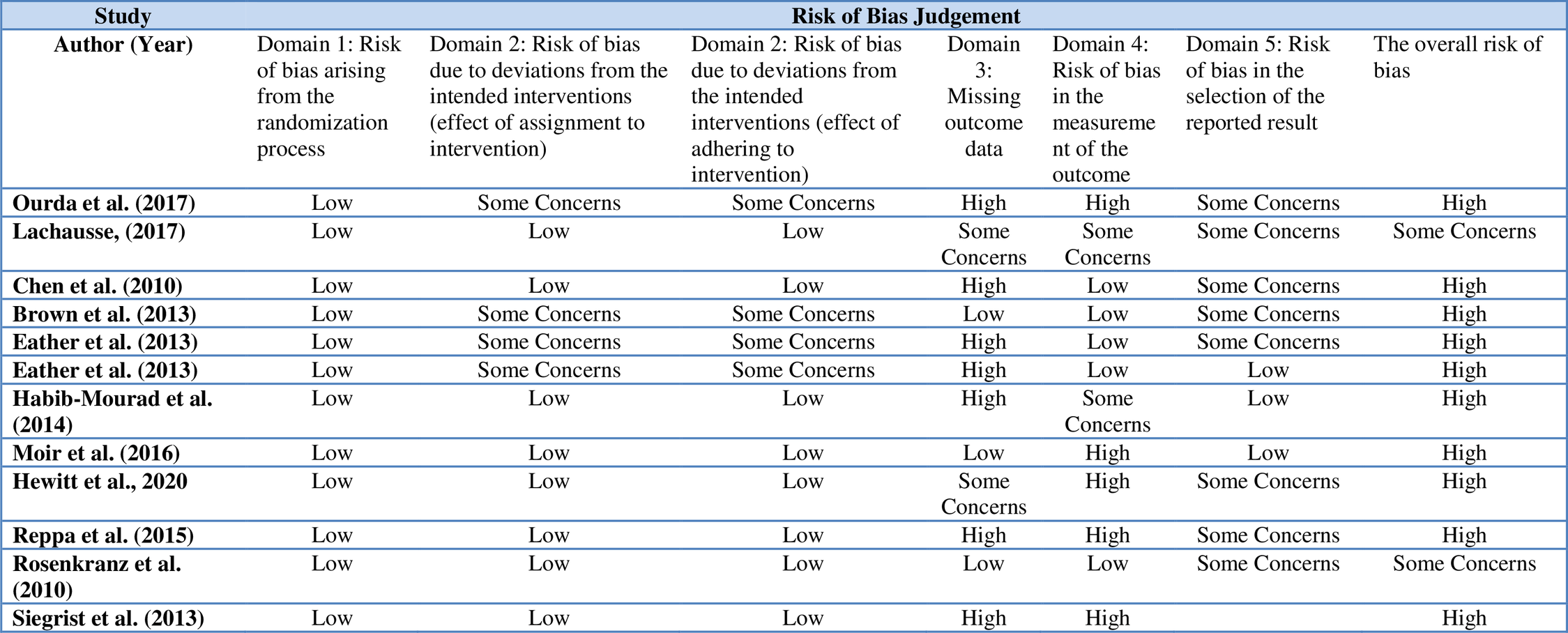

Supplement: S1 Table — (TIF) [file pone.0294987.s004.tif]
