# Supplementary material for: The effect of experiential learning interventions on physical activity outcomes in children: A systematic review
Source: PLoS One. 2023 Nov 30;18(11):e0294987. doi: 10.1371/journal.pone.0294987 (PMC10688861; doi:10.1371/journal.pone.0294987)

**S3 Fig Meta-analysis outputs for attitude outcome**

**Meta-analysis Outputs**

**
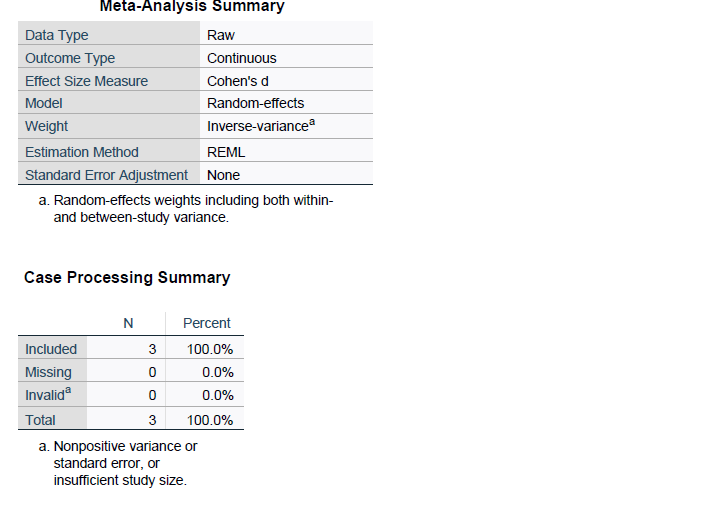
**


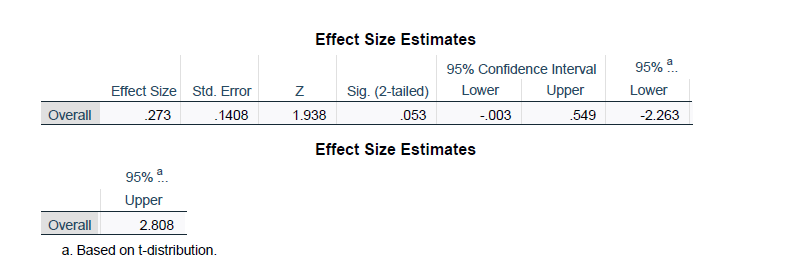


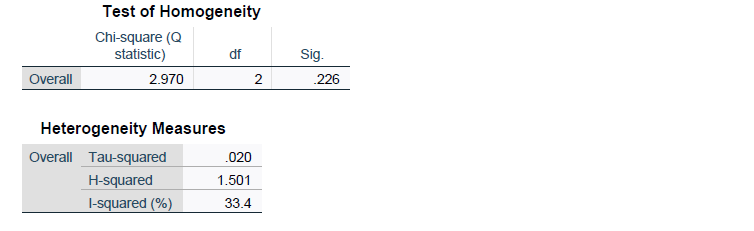


**Forest plot**


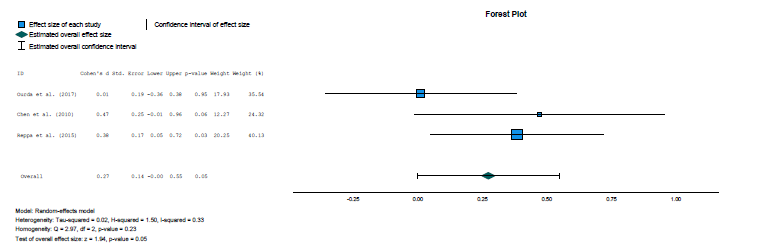


**Funnel plot**


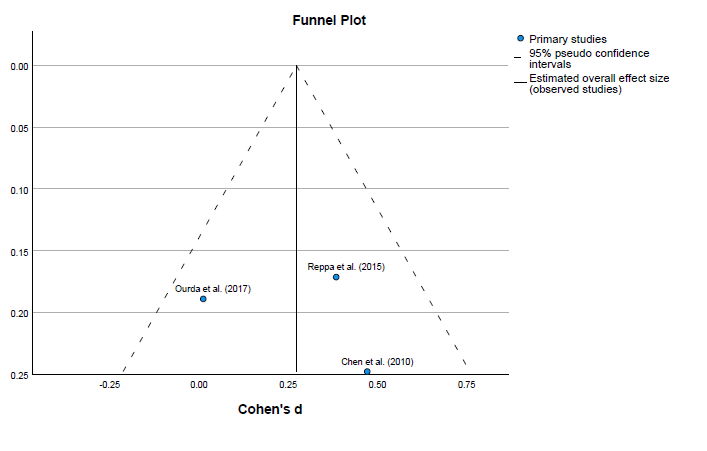

Supplement: S3 Fig — (DOCX) [file pone.0294987.s003.docx]
