# Supplementary material for: The effect of experiential learning interventions on physical activity outcomes in children: A systematic review
Source: PLoS One. 2023 Nov 30;18(11):e0294987. doi: 10.1371/journal.pone.0294987 (PMC10688861; doi:10.1371/journal.pone.0294987)
Supplement: Attachment — Submitted filename: Response to Reviewers.docx [file pone.0294987.s006.docx]

**Response to Reviewers**

Ms. Ref. No.: PONE-D-23-02390R1

Title: The effect of experiential learning interventions on physical activity outcomes in children: A systematic review

| **No.** | **Comments**  *Reviewer’s comments/questions* | **Response**  *Answer to reviewer’s comments/questions. If the text is changed to answer this question, the relevant text is inserted in the answer.* | **Reference page**  *Page and line number where change can be found.* |
| --- | --- | --- | --- |
| **Journal Requirements** | | | |
| 1) | Please review your reference list to ensure that it is complete and correct. If you have cited papers that have been retracted, please include the rationale for doing so in the manuscript text or remove these references and replace them with relevant current references. Any changes to the reference list should be mentioned in the rebuttal letter that accompanies your revised manuscript. If you need to cite a retracted article, indicate the article’s retracted status in the References list and also include a citation and full reference for the retraction notice. | The reference has been reviewed thoroughly and the DOI has been added to most of the references. | Pages 30-35  Lines 589-7797 |

| **Additional editor comments** | | | |
| --- | --- | --- | --- |
| 1) | I want to thank the authors for their revisions to this manuscript. It is already a much stronger study now. That said, I still have a number of concerns with the limited measures and interpretation of the heterogeneity and publication bias results.  First, I2 alone is not the most transparent way of reporting heterogeneity. I would expect that as a minimum, that the Q, Tau, and prediction interval statistics are reported so that the reader can determine more than simply the proportion of observed variance attributed to actual changes in effect sizes rather than sampling error. The Q statistic tests the null hypothesis of whether all studies in the model share a common effect size. The T2 statistic determines the variance of true effect sizes, while the prediction interval provides a confidence interval (with 95% confidence limits) that encompasses the range of true effect sizes for all observed samples.  Finally, Egger's regression test is only one means of reporting publications bias and my recommendation is that at least measures are used. My suggestion would be to also include the Classic Fail-Safe N or the Trim and Fill method. | Additional analyses have been conducted for heterogeneity and publication bias.  The following text has been added to the Manuscript regarding the additional tests and interpretation of the results.  Abstract- Results- Moderate to high statistical heterogeneity was observed for behaviour outcomes.  **Methods**  **Data analysis:**  The text on heterogeneity has been amended/replaced.  Possible causes of heterogeneity in effects were assessed using the I^2^ statistic, Cochran Q-tests and subgroup analyses. Cochran Q-tests were used to determine the statistical heterogeneity for physical activity behaviour and attitude outcomes, with statistical significance set at p < 0.1. All subgroup analyses used a single pooled estimate of Tau-squared (between-study variance). Random effects models were employed to allow for variation between studies. The confidence interval (with 95% confidence limits) was used to show the range of true effect sizes for included studies.  The text on publication bias has been amended/replaced.  Potential publication bias was assessed by inspecting forest and funnel plots as well as via running Egger’s Test, [39], (using a recommended criterion of p < 0.1, [40] Additionally, Duval and Tweedie’s Trim and Fill method was employed to estimate and impute potential unpublished effects to assess their impact on the pooled effect size and significance [41]. Subgroup analyses could not be performed for knowledge outcomes due to the small number of studies. Additionally, the trim and fill test for physical activity attitude outcome was not run due to not enough valid cases for processing thus no statistics were computed. The calculations for heterogeneity tests, subgroup analysis and publication bias evaluation were performed using the IBM SPSS Statistics 28.0 and the guidance from (Sen and Yildirim, 2022).  **Results:**  **Intervention effects-**  The text in the results section has been amended/replaced. We have additionally reported the results of the Tau-squared and Cochran Q tests to show heterogeneity.  The results of the heterogeneity analysis showed that there was moderate to high heterogeneity of effects across studies for physical activity behaviour outcomes. For physical activity behaviour outcome, the evidence of heterogeneity in effects was high (I^2^ = 79.9%) and (95% CI, -0.068 to -0.321), while for physical activity attitude outcome the heterogeneity in effects was low (I^2^ = 33.4%) with (95% CI, -0.003 to -0.549).  Furthermore, when the necessary analyses were performed in SPSS, the Q-statistics values (Q =36.552, df = 9, p < 0.001) were found to be statistically significant (see Fig 1 in S1 Fig).  In addition, Tau-squared, H-squared, and I-squared values were found to be 0.065, 4.985, and 79.9, respectively, showing statistically significant heterogeneity between studies for behaviour outcomes (see Fig 2 in S1 Fig).  Low heterogeneity of effects was found across no studies for attitude outcomes as shown by the Q-statistics (Q =2.970, df = 2, p < 0.226) (see Fig 2 in S1 Fig), with no statistical significance.  For behaviour outcomes, visual inspection indicates that the included studies were evenly clustered within both sides of the funnel plot, with no evidence of publication bias, (Egger’s test: t = -0.85, P < 0.4) (see Fig 3 in S1 Fig). There are not enough valid cases for processing Egger’s test for attitude outcome, so no statistics were computed (see Fig 3 in S2 Fig).  The trim and fill method suggested that no adjustments were required to correct the asymmetry of the funnel plot. Therefore, any potential publication bias was considered minimal. For physical activity attitude outcomes, there were not enough valid cases for processing thus no statistics were computed (see Fig 3 in S2 Fig).  **Discussion:**  **Limitations**- Lastly, given the moderate to high heterogeneity among the experiential learning physical activity interventions, may have distorted our conclusions reached.  **Supporting information:**  S1 Fig. Meta-analysis output for behaviour outcome.  S2 Fig. Meta-analysis output for attitude outcome. | Page 2-3  Lines 42-43  Page 10  Lines 207-212  Page 10  Lines 214-222  Page 17-18  Lines 315-336  Page 28  Lines 567-569  Page 29  Lines 595-596. |
